# NOTCH3 Is Induced in Cancer-Associated Fibroblasts and Promotes Angiogenesis in Oral Squamous Cell Carcinoma

**DOI:** 10.1371/journal.pone.0154112

**Published:** 2016-04-28

**Authors:** Kou Kayamori, Ken-ichi Katsube, Kei Sakamoto, Yoshio Ohyama, Hideaki Hirai, Akane Yukimori, Yae Ohata, Takumi Akashi, Masao Saitoh, Kiyoshi Harada, Hiroyuki Harada, Akira Yamaguchi

**Affiliations:** 1 Department of Oral Pathology, Graduate School of Medical and Dental Sciences, Tokyo Medical and Dental University, Tokyo, Japan; 2 Department of Maxillofacial Surgery, Graduate School of Medical and Dental Sciences, Tokyo Medical and Dental University, Tokyo, Japan; 3 Department of Oral and Maxillofacial Surgery, Graduate School of Medical and Dental Sciences, Tokyo Medical and Dental University, Tokyo, Japan; 4 Department of Surgical Pathology, Graduate School of Medical and Dental Sciences, Tokyo Medical and Dental University, Tokyo, Japan; 5 Department of Human Care, Tohto College of Health Science, Saitama, Japan; 6 Department of Biochemistry, Interdisciplinary Graduate School of Medicine and engineering, University of Yamanashi, Yamanashi, Japan; 7 Department of Oral Health Science Center, Tokyo dental college, Tokyo, Japan; King Faisal Specialist Hospital & Research center, SAUDI ARABIA

## Abstract

Recent studies have shown that Notch signaling is involved in many types of cancers, including oral squamous cell carcinomas (OSCCs). However, the role of Notch signaling in the tumor microenvironment is not yet fully understood. In this study, we investigated the roles of NOTCH3 signaling in cancer associated fibroblasts (CAFs) in OSCCs. Immunohistochemical study of 93 human tongue OSCC cases indicated that about one third of OSCCs showed NOTCH3 expression in CAFs, and that this expression significantly correlated with tumor-size. In vitro study showed that OSCC cell lines, especially HO1-N-1 cells stimulated NOTCH3 expression in normal human dermal fibroblasts (NHDFs) through direct cell-to-cell contact. Immunohistochemical and morphometric analysis using human OSCC samples demonstrated that NOTCH3 expression in CAFs significantly correlated with micro-vessel density in cancer stroma. In vitro angiogenesis assays involving co-culture of NHDFs with HO1-N-1 and human umbilical endothelial cells (HUVECs), and NOTCH3 knockdown in NHDFs using siRNA, demonstrated that HO1-N-1 cells significantly promoted tube formation dependent on NOTCH3-expression in NHDFs. Moreover, NOTCH3 expression in CAFs was related to poor prognosis of the OSCC patients. This work provides a new insight into the role of Notch signaling in CAFs associated with tumor angiogenesis and the possibility of NOTCH3-targeted molecular therapy in OSCCs.

## Introduction

Head and neck cancer derives from the upper aerodigestive tract including the nasal cavity, paranasal sinuses, oral cavity, pharynx and larynx. Histopathologically, the predominant malignancy in head and neck cancer is squamous cell carcinoma (SCC).

Oral SCC (OSCC) is the most common type of head and neck cancer. According to the recent GLOBOCAN estimates, approximately 300,000 new lip/oral cavity cancer patients were diagnosed in 2012 worldwide [1]. The 5-year survival rate of OSCC patients still ranges from 40 to 60% [2, 3]. Investigation regarding the molecular mechanism that regulates malignant behaviors of OSCC will be needed for development of therapeutic approaches and improvement of the poor prognosis.

Cancer stroma is composed of various types of cells including fibroblasts, immune cells, pericytes and endothelial cells. Recent studies have shown that these cells and their products establish suitable microenvironments for cancer proliferation, invasion, angiogenesis, metastasis, and chemoresistance [4, 5]. In particular, cancer-associated fibroblasts (CAFs), which are the main cancer stroma components, play a crucial role in tumor progression in various types of cancer [6]. Their origins are thought to be either tissue-resident fibroblasts, mesenchymal stem cells recruited from bone marrow, or cancer cells that underwent epithelial-mesenchymal transition [7]. Several studies have reported that CAFs stimulate cancer cell invasion [8–10] or proliferation [11] and correlate with poor prognosis in OSCCs [12, 13].

Notch signaling is an evolutionarily conserved pathway that regulates cell proliferation, apoptosis and differentiation [14]. Notch signaling is initiated by binding of NOTCH-ligand to its receptor, which is mediated by cell-to-cell contact. In humans, there are four receptors (NOTCH1-4), and five ligands (JAGGED1, 2 and DLL1, 3 and 4). Binding of the ligand to its receptor leads to cleavage and release of the intracellular domain of the NOTCH receptor (NICD). NICD translocates from the plasma membrane to the nucleus, which initiates transcription of the NOTCH target genes [15]. Recent studies have shown that dysregulation of Notch signaling is involved in diverse diseases, including various types of cancers [16, 17]. Alterations of Notch signaling in cancer cells include gain or loss of function mutations, and receptor/ligand overexpression [18]. We previously demonstrated NOTCH1 downregulation in cancer cells in OSCC by microarray and immunohistochemical studies using human OSCC samples [19], and recent studies have indicated that NOTCH1 acts as a tumor suppressor in OSCC pathogenesis [20–22].

Although both CAFs and Notch signaling play important roles in cancer progression, Notch signaling in CAFs, as opposed to cancer cells, and its contribution to malignant behavior has not been fully elucidated. NOTCH3 is physiologically expressed in the smooth muscle cells of small arteries and regulates differentiation and maturation of these cells. Loss-of-function mutation of NOTCH3 has been shown to cause cerebral autosomal dominant arteriopathy with subcortical infarcts and leukoencephalopathy (CADSIL) that is characterized by the degeneration or loss of vascular smooth muscle cells of the media, thickening of the vessel wall and deposits of granular osmiophilic materials (GOM) close to the cell surface of the smooth muscle cells or pericytes [23]. Recent studies showed that NOTCH3 is induced in fibroblasts by direct cell-to-cell contact with HUVECs and promotes vessel formation [24, 25]. These findings suggest that NOTCH3 has an essential role in the regulation of angiogenesis.

In this study, we focused on analysis of NOTCH3 in CAFs to investigate its contribution to OSCC progression. We showed NOTCH3 expression in CAFs by immunohistochemical study of samples of 93 cases of human tongue OSCC and found that NOTCH3-positive CAFs promote tumor angiogenesis in the presence of various cancer cell lines *in vitro*. Moreover, this NOTCH3 induction in CAFs correlated with poor survival of OSCC patients. These results suggest the utility of NOTCH3-targeted molecular therapy in OSCCs.

## Material and Methods

### Clinical Specimens

Primary tongue squamous cell carcinoma specimens were collected from 93 patients who had been treated at the Dental Hospital of Tokyo Medical and Dental University. Nine primary gingival SCCs, 10 buccal SCCs and 9 SCCs of floor of mouth were also collected. All of the tissues were fixed with 10% neutral buffered formalin and embedded in paraffin according to routine laboratory protocols. All experimental procedures were conducted in accordance with the amended Declaration of Helsinki and approved by the ethics committee of Tokyo Medical and Dental University (Registry No. 1063). We used surgically resected, formalin-fixed and paraffin-embedded human OSCC tissues for immunohistochemical study. Although written informed consent was not obtained from the patients, the ethics committee approved waiver of specific informed consent in accordance with amended Ethical Guidelines for Clinical Studies provided by Ministry of Health, Labor and Welfare of Japan (July 31, 2008). This research plan was disclosed in a poster format in the outpatient clinic of the oral surgery department to ensure that patients had the opportunity to decline the research use of their pathological samples, which substituted for written informed consent, and ethics committee approved this consent procedure.

### Immunostaining

For immunohistochemical staining, formalin-fixed, paraffin-embedded human OSCC tissue sections were used. The primary antibodies used were as follows: anti-NOTCH3, rabbit polyclonal, 1:200 (ab23426, Abcam, CA, USA); anti-SMA, mouse monoclonal, 1:100 (M0851, Dako, Glostrup, Sweden). Antigen retrieval was performed according to the manufacturer’s protocol. EnVision+ Dual Link (Dako) was used for secondary antibody, and coloration was conducted with diamino-benzidine substrate. For immunofluorescent double-staining, anti-NOTCH3, 1:200 (Abcam), SMA, 1:100 (mouse monoclonal, M0851, Dako or rabbit monoclonal, Clone SP171, Spring Bioscinece, CA, USA), CD34, 1:100 (mouse monoclonal, NCL-L-END, Leica Biosystems, Wetzlar, Germany) and Cytokeratin, 1:100 (mouse monoclonal, Clone AE1/AE3, M3515, Dako) were used as a primary antibody. For antigen retrieval, the sections were treated with Tris buffer (pH = 7.4) containing 0.1 mg/ml trypsin for 30 min at 37°C for CD34 antibody. Alexa Fluor 488 goat anti-rabbit IgG (A11008, Invitrogen, CA, USA) and Alexa Fluor 594 goat anti-mouse IgG (A11005, Invitrogen) were used as secondary antibody. DAPI was used for nuclear staining. The immunofluoroscent images were captured and analyzed using Axioskop2 plus microscope (Carl Zeiss, Jena, Germany).

### Evaluation of Immunohistochemical Analyses

The immunohistochemical results for NOTCH3 and α-SMA were evaluated based on the staining intensity and proportion of positive fibroblastic cells in the cancer stroma by two pathologists without prior knowledge of the patient’s clinicopathological data. Staining intensity was divided into four groups: negative, weak, moderate and strong. Cases with moderate or strong intensity in more than 30% of the fibroblasts in the cancer stroma were regarded as positive for the expression of each protein.

### Cell Culture

The following eight head and neck squamous cell carcinoma cell lines, HO1-N-1, SAS, BHY, Ca9-22, HSC3, HSC4, HSC5 and SKN3 were used. These lines were derived from the oral cavity except for HSC5 that was derived from the maxillary sinus. Six cancer cell lines were derived from non-oral lesions, HeLa from uterine cervix SCC, A549 from lung adenocarcinoma, MCF-7 from breast adenocarcinoma, MKN74 from gastric adenocarcinoma, HCT-15 from colon adenocarcinoma and Panc-1 from pancreatic adenocarcinoma, were also used. All cancer cell lines were cultured as described before [26]. HO1-N-1, SAS, Ca9-22, HSC3 and HSC5, and SKN3 were purchased from Japanese Collection of Research Bioresources (Osaka, Japan). BHY was provided by Dr. Masato Okamoto (TELLA Inc, Tokyo, Japan). HSC4 was established by Dr. Momose in the department of Oral and maxillofacial Surgery in Tokyo Medical and Dental university as described in previous report [27]. Panc-1 was purchased from American Type Culture Collection (ATCC). HeLa, A549, MKN74 and HCT-15 were purchased from RIKEN BioResource Center (Tsukuba, Japan). MCF-7 was provided by the Cell Resource Center for Biomedical Research (Tohoku University, Miyagi, Japan). Primary normal human dermal fibroblasts (NHDFs) from an adult donor were purchased from PromoCell (C-12302, Heidelberg, Germany) and primary human umbilical vein endothelial cells (HUVECs) were purchased from Lonza (CC-2517, Tokyo, Japan).

### Coculture Analyses of Cancer Cells and NHDFs

NHDFs (5×10^4^ cells/well) were seeded onto 24-well plates, incubated in Fibroblast Growth Medium 2 Kit (C-23120, PromoCell) for 48h, and then cocultured with various cancer cell lines (5×10^3^ cells/well) by incubation in α-MEM for 72h. Subsequently, the NHDFs were isolated using the following method and assessed using real-time PCR analysis. For western blot analysis, NHDFs (20×10^5^ cells/well) and cancer cell lines (2×10^4^ cells/well) were cocultured using 6-well plates.

### NHDF Isolation from Cocultures

NHDFs were isolated from cocultures by using a magnetic activated cell separation (MACS) system with anti-Fibroblast MicroBeads (130-050-601, Miltenyi Biotec, Auburn, CA, USA) and an MS column (130-042-201, Miltenyi Biotec) according to the manufacturer’s protocol.

### siRNA Transfection

Prior to coculture, NHDFs were transfected with siRNA for human NOTCH3 by using Lipofectamine 2000 (Invitrogen) according to the manufacturer’s protocol. Stealth siRNA for human NOTCH3 was purchased from Invitrogen. The sequence of the plus strand of the dsRNA was 5’-UCAAUGCUGUGGAUGAGCUUGGGAA-3’.

### Microvessel Evaluation

The microvessel density (MVD) of human OSCC samples was calculated for two groups, the SMA(+)NOTCH3(-)CAFs group and the SMA(+)NOTCH3(+)CAFs group. By using double-immunofluorescent staining for SMA or NOTCH3 and CD34, the number of CD34-positive small vessels in SMA(+)fibroblastic cancer stroma was evaluated in the former group, and the number of CD34-positive small vessels in NOTCH3(+)fibroblastic cancer stroma was counted in the latter group. Prior to counting the number of microvessels, we first examined each section at low power magnification to identify the area with highest microvessel density, and selected three fields in each case. Three micrographs at high magnification (×200) were taken. The number of CD34-positive small vessels was counted and the SMA(+)/NOTCH3(+) fibroblastic area was quantitatively analyzed using an Axioskop2 plus microscope and AxioVsion rel 4.8 software (Zeiss). Total counts of CD34-positive microvessels per total quantitated SMA(+)/NOTCH3(+) fibroblastic area of three images was calculated and defined as the MVD of each case.

### In Vitro Angiogenesis Assay

An *in vitro* endothelial-fibroblast organotypic coculture assay was modified as previously reported [28]. First (Day 0), NHDFs were seeded on 24-well plates (5.0×10^4^ cells/well) and cultured in Fibroblast Growth Medium for 48h. Then, siRNA for human NOTCH3 was transfected into the NHDFs according to the above-described method. The next day (Day 3), HO1-N-1 or A549 cancer cells (6.0×10^3^ cells/well) and HUVECs (3.0×10^4^ cells/well) were cocultured with the siRNA treated NHDFs in Endothelial cell growth medium. The culture medium was replaced every other day. On Day 9, the cells were fixed and immunohistochemically treated with the anti-CD31 antibody (mouse monoclonal, 1:100, NCL-CD31-1A10, Leica Biosystems, Newcastle, UK) and alkaline phosphatase-conjugated secondary antibody (WP20006, Invitrogen) and the signal was visualized using the BCIP/NBT substrate (11-681-45-001, Roche). Five photograph images with the highest CD31-positive capillary network in each well were captured by using an inverted microscope (ECLIPSE TS100, Nikon, Tokyo, Japan). The CD31-positive capillary network area was quantitatively analyzed using NIH image J software.

### Effect of NOTCH3 on Cell Proliferation

To examine the effect of NOTCH3 on OSCC cell line proliferation, HO1-N-1 were seeded (2.0×10^3^ cells/well) onto a 96-well plate coated with a recombinant human NOTCH3 Fc chimera (R&D Systems, Minneapolis, MN, USA). Cell proliferation was measured by using the Cell Counting Kit-8 (Dojindo, Kumamoto, Japan) according to the manufacturer’s protocol.

### Western Blotting

Western blot was conducted using standard method as described before [19]. Anti-Notch3 (D11B8, Cell Signaling, MA, USA), alpha smooth muscle Actin (ab32575, Abcam) and GAPDH (D16H11, Cell Signaling) were used as a primary antibody.

### Real-Time Quantitative PCR Analyses

RNA was extracted from NHDFs, and reverse-transcribed into cDNA as described before [26]. Real-Time PCR was performed using a Light Cycler System (Roche Diagnostics). Relative expression was calculated by the comparative C_T_ method using GAPDH as an internal control. The sequences of PCR primers were described in previous studies (*NOTCH1* and *NOTCH3* [29], *NOTCH2*, *NOTCH4* and *HEY1* [19], *α-SMA* [25], and *GAPDH* [30]).

### Statistical Analyses

Correlation between NOTCH3 expression in CAFs and clinicopathological parameters was analyzed using the chi-square or Fischer’s exact test. In *in vitro* assays, the differences in the mean values between two groups were compared using the Student’s t-test, whereas, the differences in the mean values among multiple groups were analyzed using a one-way ANOVA followed by multiple-comparison with Tukey’s methods. Overall survival rate was estimated according to the Kaplan-Meier Method and statistical significance was analyzed using the log-rank test. Overall survival times were calculated from the date of initial pathological diagnosis to the date of death. *P*-values less than 0.05 were considered statistically significant. Statistical analyses were performed using Ekuseru-Toukei 2015 (Social Survey Research Information, Tokyo, Japan).

## Results

### NOTCH3 Expression in CAFs in the Tumor Microenvironment of OSCC

**Fig 1:**
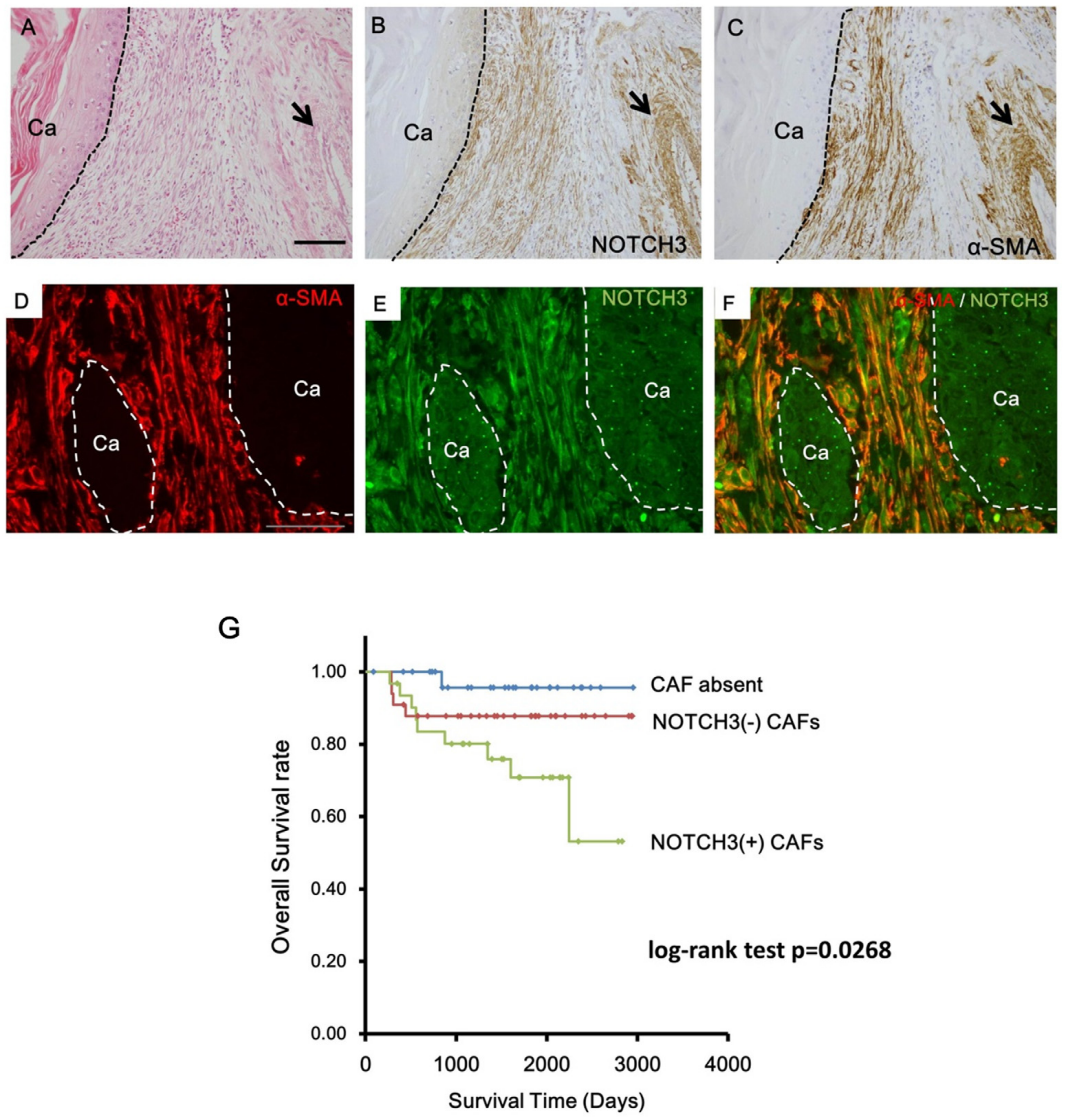
Distribution and characterization of NOTCH3-positive fibroblastic cells in cancer stroma in human OSCC. **A:** Histology of tumor invasive front. H&E staining. Scale bar, 100μm. **B:** Distribution of NOTCH3-positive fibroblastic cells. **C:** Distribution of α-SMA-positive fibroblastic cells. Cells stained brown in **B** and **C** represent positive cells for each antibody. Arrows in **A**, **B** and **C** indicate blood vessel layer, which is positive internal control of each antibody. **D**, **E**, **F:** Dual immunohistochemical analysis for α-SMA (red) and NOTCH3 (Green). Co-localization of α-SMA and NOTCH3 in fibroblasts was observed in cancer stroma. Scale bar, 50μm. Ca, cancer cells. Dotted lines in **A**-**F** indicate the interface of cancer nests and stroma. **G:** Kaplan-Meier curve for overall survival in relation to NOTCH3 expression in CAFs using 93 human OSCC cases. Log-rank test was used to calculate significance.

To investigate the distribution of NOTCH3 expressing cells in the tumor microenvironment, we immunohistochemically examined NOTCH3 expression in surgical specimens from 93 cases of tongue OSCC. Fibroblasts in cancer stroma, especially those adjacent to the tumor periphery, showed positive staining for NOTCH3 (31 out of 93 cases, 33.3%, Fig 1A and 1B). About 90% (82 out of 93 cases, 88.2%) of the cases exhibited negative or occasional weak NOTCH3 expression in the cancer cells. The latter result was compatible with our previous cDNA microarray study using human OSCC samples [19]. To further characterize the NOTCH3 positive fibroblasts, we conducted immunohistochemical staining for α-SMA, which is a marker of cancer associated fibroblasts (CAFs). Serial sections of the tumors showed that the NOTCH3-positive fibroblasts expressed α-SMA (Fig 1C). Double immunofluorecent stainig demonstrated the co-localization of NOTCH3 and α-SMA in fibroblasts (Fig 1D–1F). As summarized in Table 1, α-SMA positive fibroblasts, which could be regarded as CAFs, were observed in the cancer stroma in 64 of 93 specimens (68.8%). The remaining 29 cases showed no fibroblastic reaction around invasive cancer nests and were negative for α-SMA, although a prominent inflammatory reaction was observed. NOTCH3-positive fibroblasts that were negative for α-SMA were not observed in any case.

**Table 1:** α-SMA and NOTCH3 expression in fibroblasts in the OSCC stroma.

		NOTCH3	
Characteristics	Total	Negative, n (%)	Positive, n (%)
	**93**		
**α-SMA positive fibroblasts (CAFs)**			
**absent**	**29 (31.2%)**	**-**	**-**
**present**	**64 (68.8%)**	**33 (35.5%)**	**31 (33.3%)**

Although the most frequent involvement site for OSCC is the tongue, we further immunohistochemically investigated the NOTCH3 expression in CAFs using OSCC cases occurred at human gingiva, buccal mucosa, and floor of mouth. These results showed that NOTCH3-positive CAFs appeared in gingival SCCs (4 out of 9 cases, 44.4%), buccal SCCs (3 out of 10cases, 30%), and SCCs of the floor of mouth (3 out of 9 cases, 33.3%) (S1 Fig). There was not much difference in the frequency of NOTCH3-positive CAFs in tumor stroma among tongue SCCs and other SCCs.

These results suggested that there are OSCCs with NOTCH3-positive CAFs and OSCCs with NOTCH3-negative CAFs in their tumor stroma.

### Clinicopathological Significance of NOTCH3 Expression in CAFs in OSCCs

To characterize the significance of the presence of NOTCH3-positive CAFs in OSCC, we investigated the correlation between NOTCH3 expression in CAFs and clinico-pathological features in the above described tongue OSCC cases. As shown in Table 2, OSCCs with NOTCH3-positive CAFs are associated with significantly larger tumor size (P = 0.0292) and lymph node metastasis (P = 0.0023) compared to OSCCs without NOTCH3-positive CAFs. There was no correlation between NOTCH3 expression in CAFs and age, gender or histological differentiation of SCC. We also evaluated the correlation between NOTCH3 expression in CAFs and the prognosis of the 93 tongue OSCC patients. Kaplan-Meier survival curves demonstrated that patients with NOTCH3-positive CAFs had a poorer prognosis than those without NOTCH3-positive CAFs (Fig 1G).

**Table 2:** Clinico-pathological Significance of NOTCH3 expression in CAFs.

NOTCH3				
Characteristics	Total	Negative, n (%)	Positive, n (%)	*P*-value^a^
	64	33 (51.6%)	31 (48.4%)	
*Age*^b^(range29-79)				
≦64 years	36	16 (44.4%)	20 (55.6%)	0.218
>64years	28	17 (60.7%)	11 (39.3%)	
*Gender*				
Male	49	26 (53.1%)	23 (46.9%)	0.771
Female	15	7 (46.7%)	8 (53.3%)	
*T-stage*				
T1 (≦2cm)	19	14 (73.7%)	5 (26.3%)	0.0292*
T2, 3, 4 (>2cm)	45	19 (42.2%)	26 (57.8%)	
*N-stage*				
N0	45	29 (64.4%)	16 (35.6%)	0.0023**
≧N1	19	4 (21.1%)	15 (78.9%)	
*Differentiation*				
well	27	15 (55.6%)	12 (44.4%)	0.442
moderate	19	11 (57.9%)	8 (42.1%)	
poorly	18	7 (38.9%)	11 (61.1%)	

^a^ *P* values were calculated by the Fischer’s exact test. Only ‘Differentiaton’ was calculated by chi-square test.

*P < 0.05,

**P < 0.01

^b^ Median years

### Human OSCCs Stimulate NOTCH3 Expression in Fibroblasts

**Fig 2:**
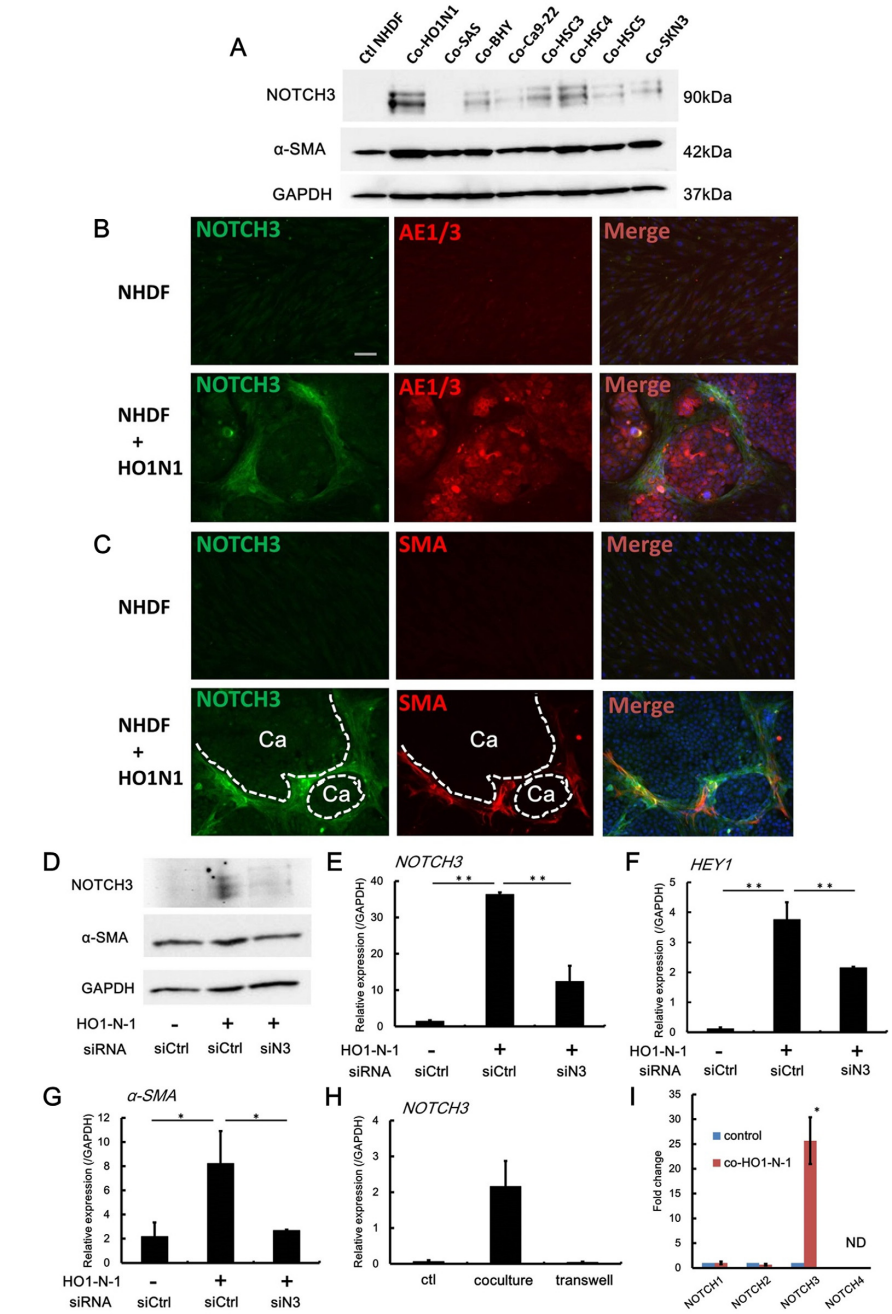
OSCC stimulates NOTCH3 expression in fibroblasts. **A:** NHDFs were cultured alone (ctl; control) or cocultured with represented oral and maxillary SCC cell lines. 3 days after coculture, NHDFs were isolated from this coculture by using anti-Fibroblast microbeads for Western blot analysis. **B and C:** Immunofluorostainig using coculture with HO1-N-1 and NHDFs. Culture of NHDFs alone was used as a control. The bundles of NOTCH3-positive NHDFs (green) were observed around the AE1/AE3-positve HO1-N-1 cancer nests (red) (**B**). Double NOTCH3 and α-SMA-positive NHDFs were intervened between HO1-N-1 cancer nests. Ca, HO1-N-1 cancer nests. Dotted lines demonstrate the interface of HO1-N-1 cancer nests and NHDFs. (**C**). Scale bar, 100μm. The nuclei were counterstained by DAPI. **D-G:** NHDFs transfected with negative control siRNA (siCtrl) or siRNA for NOTCH3 (siN3) was cultured alone or cocultured with HO1-N-1 cells. 3 days after coculture, NHDFs were isolated from this coculture and subjected to western blot (**D**) and qPCR analyses to measure NOTCH3 (**E**), HEY1 (**F**), α-SMA (**G**). **H:** NHDFs were cultured alone (control), or directly cocultured with HO1-N-1 cells, or cocultured with transwell-separated HO1-N-1 cells. 3 days after culture, NHDFs were isolated and subjected to qPCR analysis. **I:** qPCR to measure each NOTCH mRNA expression in NHDFs isolated from coculture with HO1-N-1 cells. ND, not detected. *P < 0.05, **P < 0.01.

To confirm these immunohistochemical results, we performed *in vitro* experiments using cocultured OSCC cell lines and primary normal human dermal fibroblasts (NHDFs). Following co-culture, the NHDFs were separated using anti-Fibroblasts Microbeads as described in a previous report [31]. Western blot analysis of these NHDFs indicated that their coculture with OSCCs, especially with HO1-N-1 cells, stimulated NOTCH3 expression in the NHDFs compared to the basal expression when the NHDFs were cultured alone (Fig 2A). Moreover, α-SMA protein levels were also higher in NHDFs cocultured with OSCCs compared to control NHDFs (Fig 2A). We then further analyzed NHDF/OSCC co-culture using HO1-N-1 cells and NHDFs. Double immunofluorescent staining of NOTCH3 and keratin in this coculture model showed that bundles of NOTCH3-positive NHDFs surrounded keratin-positive HO1-N-1 cancer nests (Fig 2B), which resembled the distribution of NOTCH3-positive fibroblasts around the cancer nests in the human OSCC samples. Moreover, double immunofluorescent staining for NOTCH3 and α-SMA showed that these NOTCH3-positive NHDFs also co-expressed α-SMA (Fig 2C). Western blotting and quantitative real-time PCR analysis of the NHDFs separated from this coculture showed that NOTCH3 expression was significantly upregulated at both the protein and the mRNA level in the NHDFs cocultured with HO1-N-1, compared to the NHDFs cultured alone (Fig 2D and 2E; siCtrl). The mRNA expression of HEY1, a downstream target of NOTCH, was also significantly increased in NHDFs co-cultured with HO1-N-1 compared to its expression in the absence of HO1-N-1 (Fig 2F; siCtrl). Additionally, α-SMA expression in NHDFs cocultured with HO1-N-1 was also increased at both the protein and mRNA levels compared to culture in the absence of HO1-N-1 (Fig 2D–2F; siCtrl). The induction of HEY1 and α-SMA by coculture was significantly inhibited in NHDFs transfected with siRNA for NOTCH3 (siN3) compared to cells transfected with control siRNA (siCtrl) (Fig 2D, 2F and 2G). This siN3 significantly suppressed NOTCH3 expression compared to siCtrl (Fig 2D and 2E). Collectively, these data indicate that OSCCs have the potential to induce NOTCH activity by stimulating the expression of NOTCH3 in NHDFs, and that NOTCH3 expression regulates α-SMA expression. The above NOTCH3 induction in NHDFs was observed when HO1-N-1 and NHDFs were cocultured in a direct contact system. Separation co-culture using a porous transwell did not induce upregulation of *NOTCH3* expression in NHDFs (Fig 2H). This result implies that juxtacrine signaling through cell-to-cell contact between cancer cells and fibroblasts is essential for the induction of NOTCH3 expression in fibroblasts. Moreover, the mRNA expression level of NOTCH1 or NOTCH2 in NHDFs cocultured in a direct contact system with HO1-N-1 cells showed no significant change compared to the NHDFs cultured alone. NOTCH4 expression was not detected in this system (Fig 2I). These results suggest that OSCCs specifically stimulate NOTCH3 expression in fibroblasts.

### NOTCH3 Expressed in CAFs Regulates Angiogenesis

**Fig 3:**
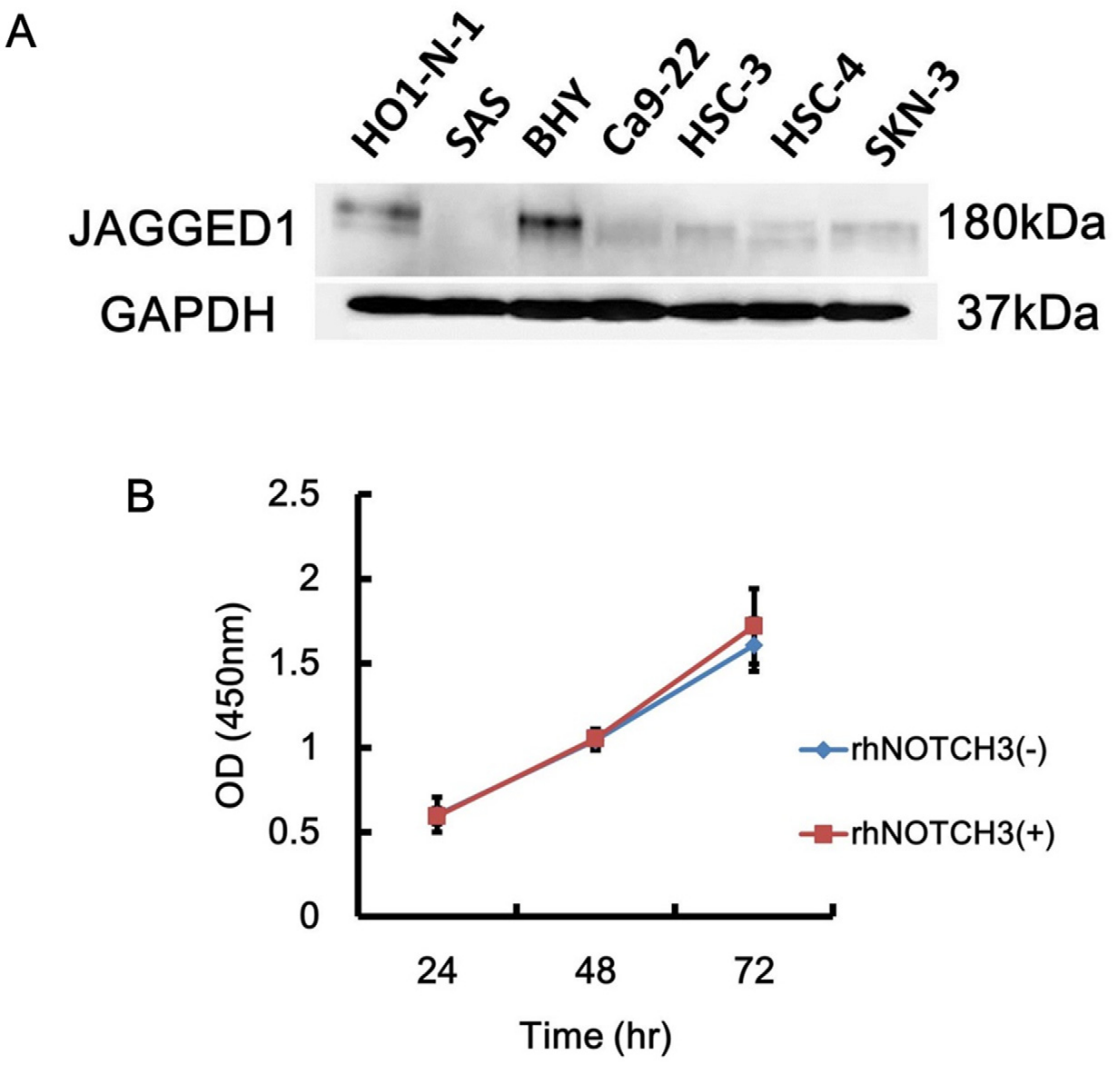
NOTCH3 did not directly affect OSCC cells proliferation. **A:** JAGGED1 expression in various oral cancer cell lines was analyzed by Western blot. **B:** The effect of NOTCH3 on cell proliferation. The proliferation of HO1-N-1 cells with or without recombinant human NOTCH3-Fc chimera treatment was monitored for 72hr.

The immunohistochemical study using human OSCC samples indicated that NOTCH3 expression in CAFs significantly correlated with T-stage (tumor size). This result suggested that NOTCH3-positive CAFs promote tumor growth by stimulating tumor cell proliferation and/or by enhancing angiogenesis. To investigate these possibilities, we first performed an *in vitro* cell proliferation assay using HO1-N-1 cells, which expresses the NOTCH ligand JAG1 as determined by Western blotting (Fig 3A). Treatment with a recombinant human NOTCH3-Fc/chimera, which can bind to its ligand, JAG1, did not affect HO1-N-1 proliferation (Fig 3B).

**Fig 4:**
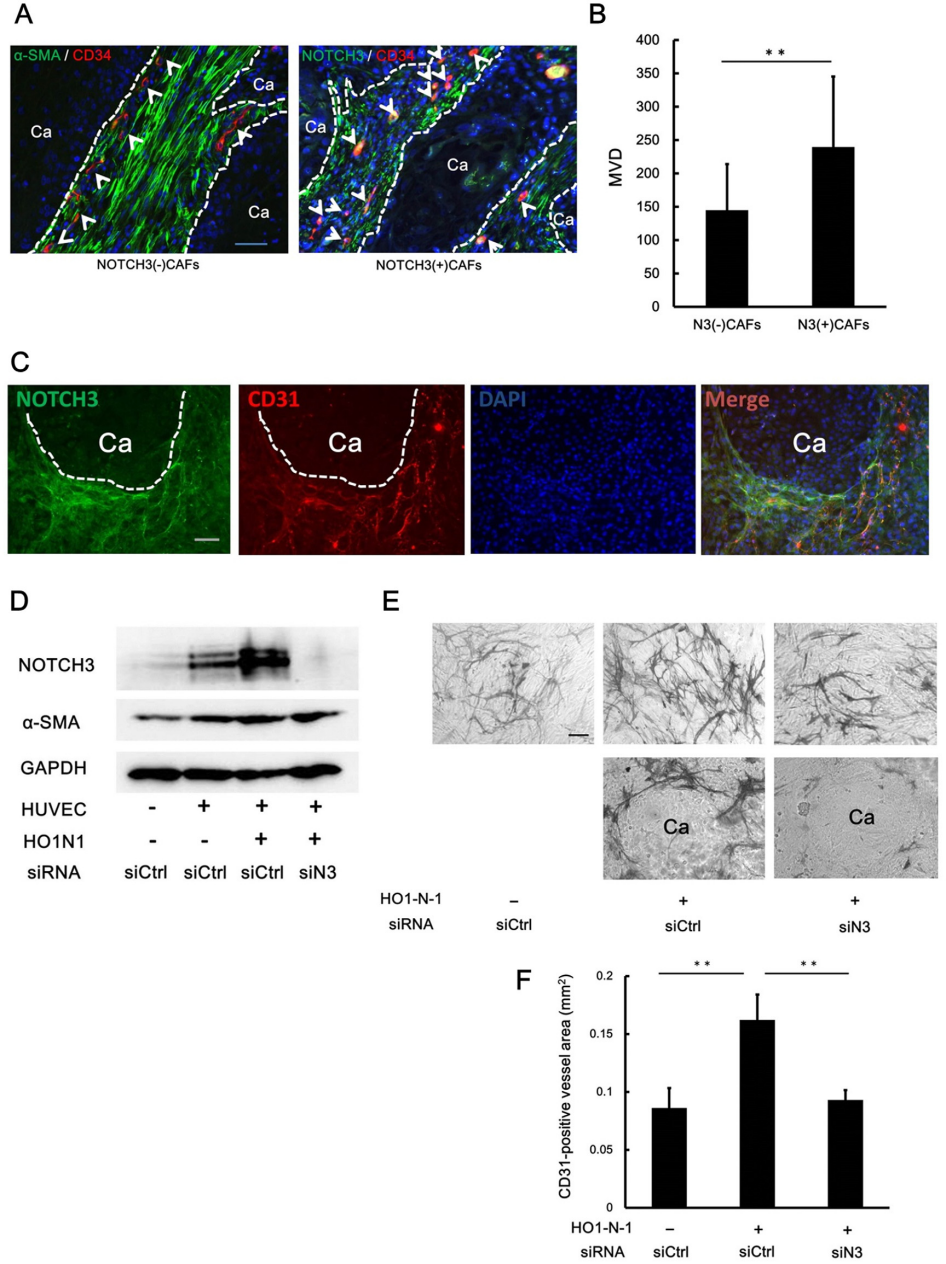
NOTCH3-positive CAFs promotes angiogenesis. **A and B**: Comparison of the microvessel density (MVD) between NOTCH3(-) CAFs and NOTCH3(+)CAFs cases. Immunofluorostaining for α-SMA (green), NOTCH3 (green) and CD34 (red) using human tongue OSCC samples. Ca, cancer nests. Dotted lines showed the interface of the cancer nests and stroma. Arrows demonstrated the CD34-positive microvessel. Scale bar, 50μm. The nuclei were counterstained by DAPI (**A**). Quantitative evaluation of MVD between these two groups (**B**). **C-F**: *In vitro* angiogenesis assay cocultured with HO1-N-1 cells, HUVECs and siRNA transfected NHDFs. Immunofluorostaining for NOTCH3 (green) and CD31 (red). Ca, HO1-N-1 cancer nests. Dotted lines indicate the margin of the HO1-N-1 cancer nests. Scale bar, 100μm. The nuclei were counterstained by DAPI (**C**). NHDFs isolated from this coculture were subjected to Western blot (**D**). Representative image of tube formation by HUVECs in each condition. Scale bar, 100μm (**E**). Quantitative evaluation of tube formation area (CD31-positive area) in each condition (**F**). siCtrl, negative control siRNA. siN3, siRNA for NOTCH3. **P < 0.01.

Next, we conducted double immunofluorescent staining of NOTCH3 and CD34 (a marker of endothelial cells) to determine if NOTCH3 expression in CAFs is associated with the density of blood vessels in human tongue OSCC samples. Micro-vessel density at the tumor invasive area was significantly increased in the NOTCH3(+)CAFs group compared to the NOTCH3(-)CAFs group (Fig 4A and 4B). This result suggested that NOTCH3(+)CAFs promote angiogenesis. To further investigate the mechanism of such angiogenesis, we performed an organotypic angiogenesis assay by coculturing HO1-N-1 cells, HUVEC and NHDFs to determine whether OSCC-induced NOTCH3 in NHDFs facilitates vessel formation. Immunofluorescent staining of this co-culture showed a CD31-positive meshwork formed by HUVECs and NOTCH3-positive NHDFs around the cancer cell nests (Fig 4C). Western blot analysis indicated that NHDFs cocultured with HUVECs alone, in the absence of HO1-N-1 cells showed NOTCH3 induction compared to the control NHDFs. Much higher levels of NOTCH3 expression were observed in NHDFs that were cocultured with HUVECs together with HO1-N-1 cells (Fig 4D). To evaluate tube formation in this assay, we visualized CD31-positive cells by immunostaining. Compared to the control group, CD31–positive tube formation by HUVECs in the presence of NHDFs and control siRNA was significantly increased by co-culture with HO1-N-1. This tube formation was significantly suppressed when the NHDFs were transfected with siN3 (Fig 4E and 4F). These results suggested that OSCCs promote angiogenesis by inducing NOTCH3 expression in CAFs.

**Fig 5:**
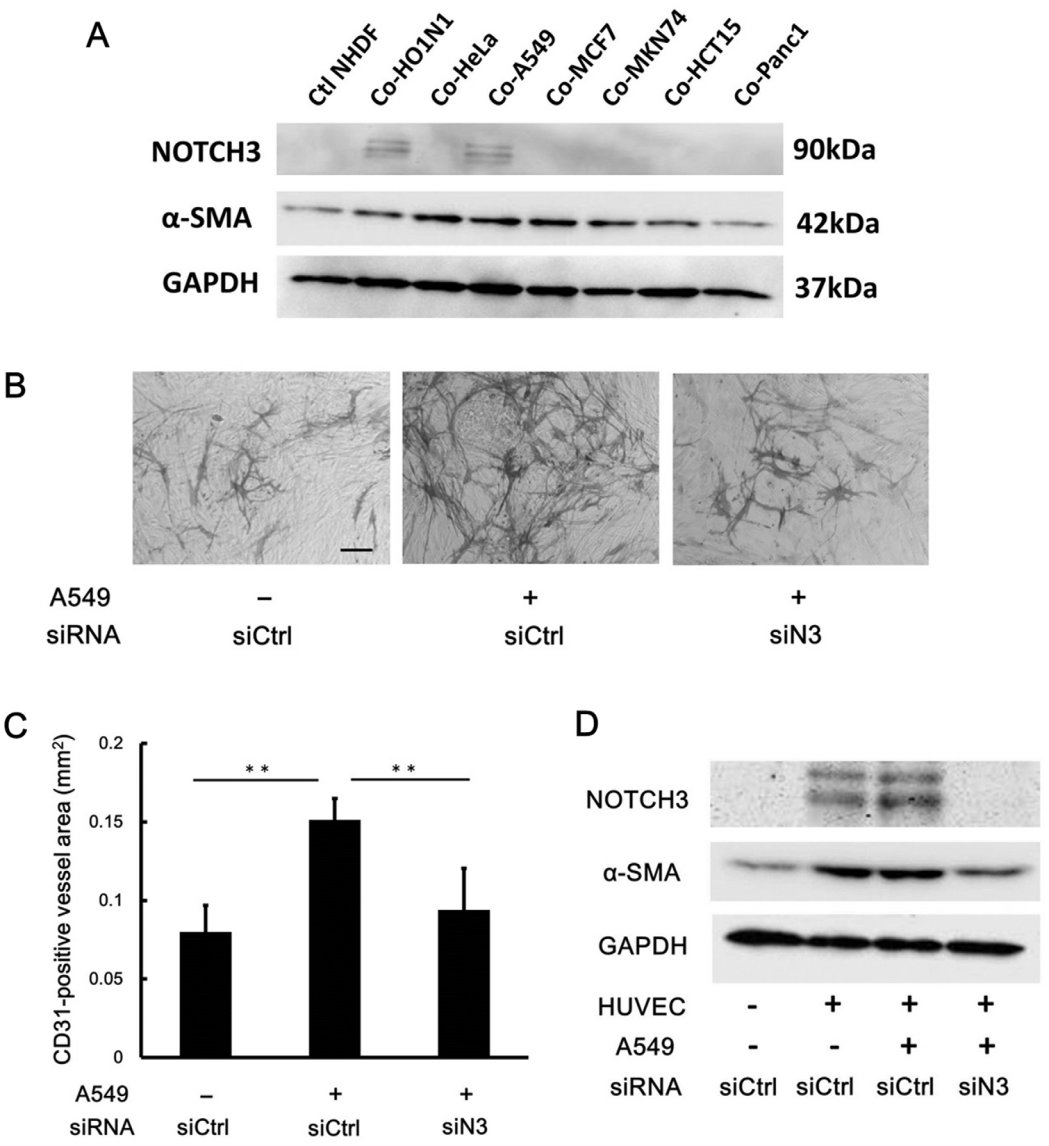
A549 promotes NOTCH3 expression in NHDFs and angiogenesis *in vitro*. **A:** NHDFs were cocultured with various cancer cell lines derived from non-oral lesions. 3 days after coculture, NHDFS were isolated from this coculture and subjected to Western blot analysis to detect NOTCH3 and α-SMA. **B-D:** In vitro angiogenesis assay cocultured with A549, HUVECs and siRNA transfected NHDFs. Representative images of tube formation (**B**) and its quantitative evaluation (**C**) in each condition. Scale bar, 100μm. NHDFs isolated from this angiogenesis assay were subjected to Western blot analysis (**D**). siCtrl, negative control siRNA. siN3, siRNA for NOTCH3. **P < 0.01.

To investigate whether NOTCH3 induction in fibroblasts is a unique property of OSCCs, we cocultured NHDFs with cell lines derived from cancers of various organs including uterine cervix, lung, breast, stomach, colon, and pancreas. Western blotting analysis indicated that A549 cells, which are a lung adenocarcinoma cell line, stimulated the expression of NOTCH3 in NHDFs at a level that was similar to that induced by HO1-N-1 (Fig 5A). An *in vitro* angiogenesis assay showed that A549 cells significantly induced tube formation in HUVECs when co-cultured with NHDFs and that this meshwork was significantly reduced by NOTCH3 knockdown in NHDFs (Fig 5B and 5C). Western blot analysis of NHDFs isolated from the angiogenesis assay revealed similar results to those obtained when NHDFs cocultured with HUVECs and HO1-N-1 were analyzed (Fig 5D). These results indicate that cancer cells other than OSCC can induce NOTCH3 in CAFs and promote angiogenesis.

**Fig 6:**
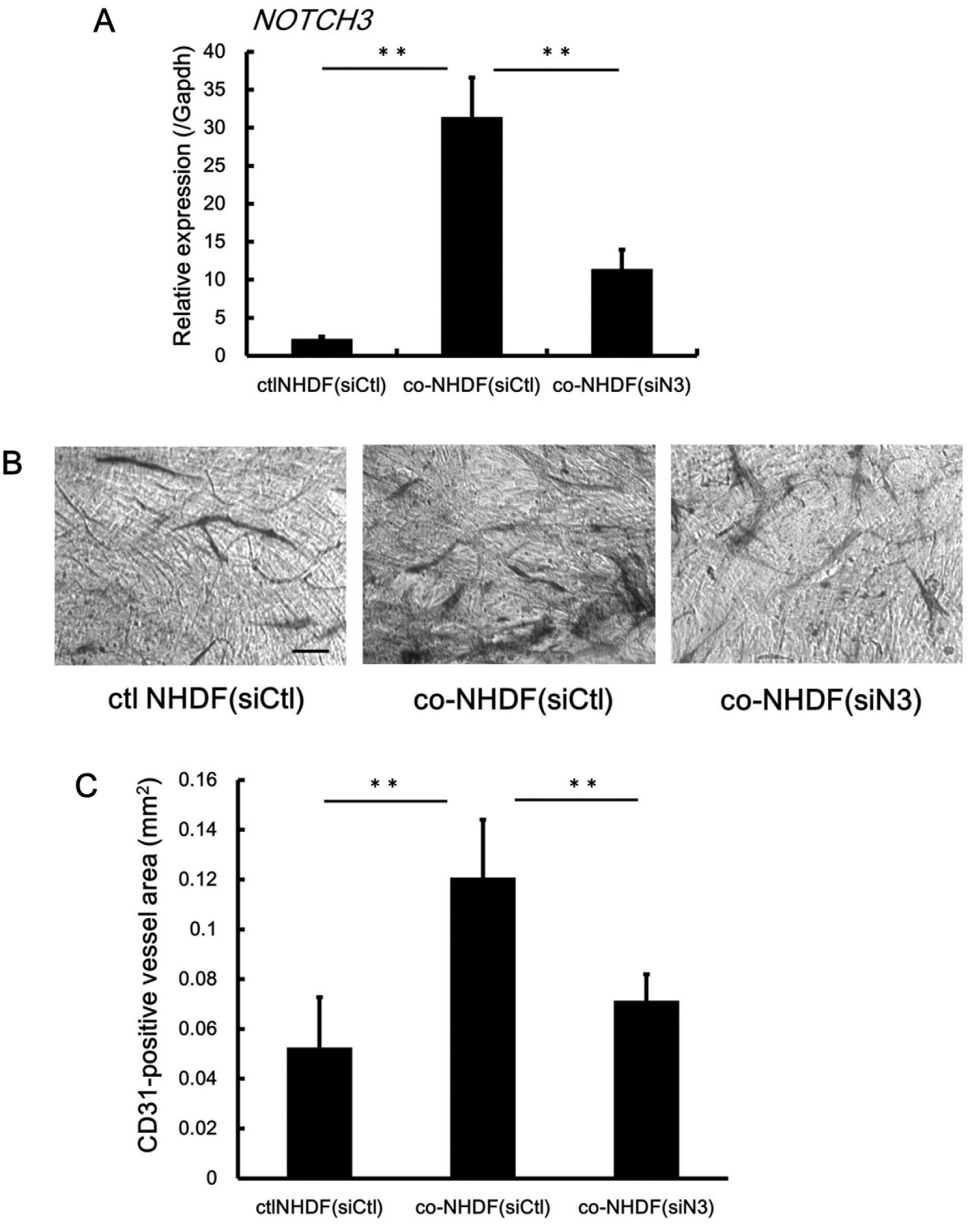
NHDFs isolated from coculture with HO1-N-1 promotes angiogenesis *in vitro*. **A:** NHDFs were cocultured with HO1-N-1 for 3 days. Prior to coculture, NHDFs were transfected with negative control siRNA or siRNA for NOTCH3. After 3 days, NHDFs were isolated from this coculture by using anti-fibroblast microbeads. NHDFs were subjected to real-time qPCR analyses to measure Notch3 expression. **B-C:** These isolated NHDFs were seeded on 24-well plates (2.5×10^5^ cells/well) and cocultured with HUVECs (3.0×10^4^ cells/well) for 4 days. Representative images of tube formation (**B**) and its quantitative evaluation (**C**) in each condition. Scale bar, 100μm. ctlNHDF(siCtl), negative control siRNA transfected NHDF isolated from culture alone. co-NHDF(siCtl), negative control siRNA transfected NHDF isolated from coculture with HO1-N-1. co-NHDF(siN3), siRNA for NOTCH3 transfected NHDF isolated from coculutre with HO1-N-1. **P < 0.01.

Finally, to further characterize the pro-angiogenic effect of NOTCH3-expressing NHDFs, we isolated NHDFs from coculture with HO1-N-1 (Fig 6A). These activated NHDFs were then cocultured with HUVECs in absence of cancer cells for 4days and the tube formation was evaluated. Compared to the control group, tube formation significantly increased in NHDFs isolated from coculture with HO1-N-1. Moreover, this tube formation significantly decreased in siN3 transfected NHDFs isolated from coculture with HO1-N-1 (Fig 6B and 6C).

## Discussion

Angiogenesis, which is the formation of new blood vessels from pre-existing ones, plays an important role in cancer proliferation. Moreover, angiogenesis aids cancer cells to escape from the primary site into the blood stream and to establish distant metastasis in a secondary organ. This complex process is regulated by various angiogenic factors produced by the tumor microenvironment including by tumor cells, fibroblasts and macrophages. However, the contribution of CAFs to angiogenesis is not yet fully understood.

Mural cells, such as pericytes and vascular smooth muscle cells, are located at the interface of the endothelium and surrounding stromal tissues. Pericytes interact with endothelial cells and regulate blood flow, vessel permeability, stabilization, remodeling and maturation of blood vessels [32, 33]. In addition to these functions, pericytes contribute to angiogenesis under physiological and various pathological conditions [34]. Pericytes play a pivotal role in supporting angiogenesis by regulating the migration, growth, maturation and stabilization of endothelial cells [34, 35]. Recent studies demonstrated that Notch signaling between mural cells and endothelial cells is an important pathway for vascular formation [36, 37]. Lilly B et al performed microarray analysis to identify the genes that are involved in angiogenesis using a three-dimensional coculture model of HUVECs and human dermal fibroblasts. They discovered that *NOTCH3* is a candidate angiogenic gene, whose expression was substantially increased in fibroblasts when cocultured with HUVECs [24]. Liu H et al reported that this NOTCH3 induction stimulates the expression of α-SMA in human dermal fibroblasts and modulates vessel formation by HUVECs [25]. Our study showed that OSCC cell lines stimulated NOTCH3 and α-SMA expression in fibroblasts and that this α-SMA induction was partially regulated by NOTCH3 activity. Moreover, these NOTCH3-overexpressing fibroblasts had the potential to induce vessel formation by HUVECs. Although the detailed mechanism of vessel formation induction by NOTCH3 signaling has not been fully clarified, an *in vivo* study using *Notch3*-null mice demonstrated that NOTCH3 in mural cells regulates vessel sprouting in developing retinal capillaries [38]. These results suggested the possibility that NOTCH3(+)CAFs might positively regulate vessel sprouting, which would result in an overall increase in vessel density. Based on the combined data, we assume that CAFs in OSCCs ‘mimic’ the function of mural cells through induction of NOTCH3, which leads to the promotion of tumor angiogenesis.

Notch signaling functions as an angiogenic regulator in physiological and pathological conditions, including cancer. Sprouting angiogenesis involves two distinct endothelial cell types, namely tip and stalk cells, and the Notch pathway plays an important role in specification of these two cells. In response to vascular endothelial growth factor (VEGF), tip cells, which are located at the leading edge of the vessels, produces the NOTCH ligand DLL4. DLL4 then binds to NOTCH1 on neighboring endothelial cells, which causes these cells to become stalk cells and adopt a proliferative behavior for vessel elongation [39, 40]. NOTCH3 is predominantly expressed on mural cells and interacts with endothelial JAG1. This heterotypic communication leads to stabilization and maturation of vessels [25, 36, 37]. NOTCH3(+)CAFs may promote sprouting of tumor endothelial cells by a molecular mechanism similar to that of the DLL4/NOTCH1 pathway and stabilize the newly formed tumor vessels through JAG1/NOTCH3 interactions between endothelial cells and CAFs.

Our study demonstrated that NOTCH3 induction in fibroblasts needs cell-to-cell contact between cancer cells and fibroblasts. We presume that juxtacrine or paracrine factors that exert their influence over a short distance may play an important role in this NOTCH3 induction. Immunohistochemical analysis using surgically excised OSCC samples showed that NOTCH3-positive CAFs were located not only close to the periphery of cancer nests but also at some distance from the cancer nests. These results imply that paracrine as well as juxtacrine mechanisms participate in the NOTCH3 induction in CAFs. Notch signaling is known to mediate lateral induction of the signal, in which a ligand-expressing cell stimulates neighboring cells to upregulate ligand expression, thereby causing the propagation of Notch signaling activity. Notch-mediated lateral induction is observed in the development of various organs [41]. Previous studies of arterial wall formation have shown that initial heterotypic interaction between JAG1-expressing endothelial cells and NOTCH3-expressing smooth muscle cells stimulates JAG1 expression in smooth muscle cells, which then leads to propagation of JAG1 expression in the neighboring smooth muscle cells by lateral induction to promote differentiation of these cells [38, 42]. Moreover, Liu H et al transfected an active form of human NOTCH3 (NICD3) into fibroblasts and found that NICD3 significantly induces NOTCH3 and JAG1 expression in fibroblasts. This result implies that NOTCH3 can autoregulate its own expression [25]. These studies suggest one possible mechanism by which NOTCH3 expression could be propagated towards the CAFs that are far from the cancer nests i.e., direct interaction of NOTCH3 with JAG1-expressing OSCCs might initiate the induction of NOTCH3 expression in CAFs. These CAFs might then induce JAG1 and NOTCH3 expression in neighboring CAFs. Another possible explanation of the propagation of NOTCH3-expressing CAFs is through the action of paracrine factors. Thus, cell-to-cell contact between cancer cells and CAFs may lead to the production of paracrine factors that can stimulate NOTCH3 expression in CAFs. Further analysis will be required to determine the details of the mechanism for the propagation of NOTCH3 signaling to distant CAFs.

Previous studies have shown that NOTCH3 is overexpressed in the tumor cells of various types of cancers, including non-small cell lung cancers, pancreatic cancers, colorectal cancers and ovarian cancers, and that such overexpression correlates with poor prognosis of the patients [43–46]. Zhang et al reported that NOTCH3 expression in cancer cells of OSCCs significantly correlated with clinical stage [47]. However, the relationship between NOTCH3 expression and prognosis in OSCCs has not been reported. In the present study, we focused on NOTCH3 expression in CAFs, not in tumor cells, and demonstrated that the expression of NOTCH3 in CAFs is associated with poor prognosis of OSCC patients. These data suggest that NOTCH3 expression in CAFs might be used as a prognostic marker of OSCCs.

In this study, we demonstrated that OSCCs stimulated the surrounding CAFs to express NOTCH3 in a cell-to-cell contact dependent manner, which activated the CAFs and resulted in their involvement in angiogenesis, thereby promoting tumor growth. Targeting NOTCH3-positive CAFs may provide a new therapeutic approach for OSCC treatment. Tarextumab is a human monoclonal antibody that targets NOTCH2 and NOTCH3 and inhibits their functions. Tarextumab has recently tested in a phase II clinical trial for the treatment of pancreatic adenocarcinoma and pulmonary small cell carcinoma, which exhibit dysregulated NOTCH2 and/or NOTCH3 activity in tumor cells [48]. Employment of a similar molecular targeted drug against the Notch signaling pathway in OSCC treatment will be useful for blocking excessive NOTCH3 activity in CAFs, which would suppress tumor angiogenesis.

## Supporting Information

S1 Fig NOTCH3-positive CAFs in tumor stroma in human gingival, buccal and floor of mouth SCCs. Immunohistochemical analyses for α-SMA and NOTCH3 in human gingival, buccal and floor of mouth SCC samples. Scale bar, 100μm. Ca, cancer cells.
(TIF)

## References

[1] Chi AC, Day TA, Neville BW. Oral cavity and oropharyngeal squamous cell carcinoma-an update. CA Cancer J Clin. 2015. 10.3322/caac.21293 26215712

[2] Gorsky M, Epstein JB, Oakley C, Le ND, Hay J, Stevenson-Moore P. Carcinoma of the tongue: a case series analysis of clinical presentation, risk factors, staging, and outcome. Oral Surg Oral Med Oral Pathol Oral Radiol Endod. 2004;98(5):546–52. 15529126 10.1016/j.tripleo.2003.12.041

[3] Jemal A, Siegel R, Ward E, Hao Y, Xu J, Thun MJ. Cancer statistics, 2009. CA Cancer J Clin. 2009;59(4):225–49. 10.3322/caac.20006 19474385

[4] Sun Y. Translational horizons in the tumor microenvironment: harnessing breakthroughs and targeting cures. Medicinal research reviews. 2015;35(2):408–36. 10.1002/med.21338 25588753 PMC4374701

[5] Castells M, Thibault B, Delord JP, Couderc B. Implication of tumor microenvironment in chemoresistance: tumor-associated stromal cells protect tumor cells from cell death. Int J Mol Sci. 2012;13(8):9545–71. 10.3390/ijms13089545 22949815 PMC3431813

[6] Kalluri R, Zeisberg M. Fibroblasts in cancer. Nat Rev Cancer. 2006;6(5):392–401. 16572188 10.1038/nrc1877

[7] Madar S, Goldstein I, Rotter V. 'Cancer associated fibroblasts'—more than meets the eye. Trends Mol Med. 2013;19(8):447–53. 10.1016/j.molmed.2013.05.004 23769623

[8] Daly AJ, McIlreavey L, Irwin CR. Regulation of HGF and SDF-1 expression by oral fibroblasts—implications for invasion of oral cancer. Oral Oncol. 2008;44(7):646–51. 17996483 10.1016/j.oraloncology.2007.08.012

[9] Sobral LM, Bufalino A, Lopes MA, Graner E, Salo T, Coletta RD. Myofibroblasts in the stroma of oral cancer promote tumorigenesis via secretion of activin A. Oral Oncol. 2011;47(9):840–6. 10.1016/j.oraloncology.2011.06.011 21727023

[10] Fullar A, Kovalszky I, Bitsche M, Romani A, Schartinger VH, Sprinzl GM, et al Tumor cell and carcinoma-associated fibroblast interaction regulates matrix metalloproteinases and their inhibitors in oral squamous cell carcinoma. Experimental cell research. 2012;318(13):1517–27. 10.1016/j.yexcr.2012.03.023 22516051 PMC3378977

[11] Kellermann MG, Sobral LM, da Silva SD, Zecchin KG, Graner E, Lopes MA, et al Mutual paracrine effects of oral squamous cell carcinoma cells and normal oral fibroblasts: induction of fibroblast to myofibroblast transdifferentiation and modulation of tumor cell proliferation. Oral Oncol. 2008;44(5):509–17. 17826300 10.1016/j.oraloncology.2007.07.001

[12] Bello IO, Vered M, Dayan D, Dobriyan A, Yahalom R, Alanen K, et al Cancer-associated fibroblasts, a parameter of the tumor microenvironment, overcomes carcinoma-associated parameters in the prognosis of patients with mobile tongue cancer. Oral Oncol. 2011;47(1):33–8. 10.1016/j.oraloncology.2010.10.013 21112238

[13] Fujii N, Shomori K, Shiomi T, Nakabayashi M, Takeda C, Ryoke K, et al Cancer-associated fibroblasts and CD163-positive macrophages in oral squamous cell carcinoma: their clinicopathological and prognostic significance. J Oral Pathol Med. 2012;41(6):444–51. 10.1111/j.1600-0714.2012.01127.x 22296275

[14] Artavanis-Tsakonas S, Rand MD, Lake RJ. Notch signaling: cell fate control and signal integration in development. Science. 1999;284(5415):770–6. 10221902 10.1126/science.284.5415.770

[15] Kopan R, Ilagan MX. The canonical Notch signaling pathway: unfolding the activation mechanism. Cell. 2009;137(2):216–33. 10.1016/j.cell.2009.03.045 19379690 PMC2827930

[16] Ranganathan P, Weaver KL, Capobianco AJ. Notch signalling in solid tumours: a little bit of everything but not all the time. Nat Rev Cancer. 2011;11(5):338–51. 10.1038/nrc3035 21508972

[17] Penton AL, Leonard LD, Spinner NB. Notch signaling in human development and disease. Semin Cell Dev Biol. 2012;23(4):450–7. 10.1016/j.semcdb.2012.01.010 22306179 PMC3638987

[18] Ntziachristos P, Lim JS, Sage J, Aifantis I. From fly wings to targeted cancer therapies: a centennial for notch signaling. Cancer Cell. 2014;25(3):318–34. 10.1016/j.ccr.2014.02.018 24651013 PMC4040351

[19] Sakamoto K, Fujii T, Kawachi H, Miki Y, Omura K, Morita K, et al Reduction of NOTCH1 expression pertains to maturation abnormalities of keratinocytes in squamous neoplasms. Lab Invest. 2012;92(5):688–702. 10.1038/labinvest.2012.9 22330335

[20] Pickering CR, Zhang J, Yoo SY, Bengtsson L, Moorthy S, Neskey DM, et al Integrative genomic characterization of oral squamous cell carcinoma identifies frequent somatic drivers. Cancer Discov. 2013;3(7):770–81. 10.1158/2159-8290.CD-12-0537 23619168 PMC3858325

[21] Agrawal N, Frederick MJ, Pickering CR, Bettegowda C, Chang K, Li RJ, et al Exome sequencing of head and neck squamous cell carcinoma reveals inactivating mutations in NOTCH1. Science. 2011;333(6046):1154–7. 10.1126/science.1206923 21798897 PMC3162986

[22] Brakenhoff RH. Cancer. Another NOTCH for cancer. Science. 2011;333(6046):1102–3. 10.1126/science.1210986 21868662

[23] Joutel A. Pathogenesis of CADASIL: transgenic and knock-out mice to probe function and dysfunction of the mutated gene, Notch3, in the cerebrovasculature. Bioessays. 2011;33(1):73–80. 10.1002/bies.201000093 20967782

[24] Lilly B, Kennard S. Differential gene expression in a coculture model of angiogenesis reveals modulation of select pathways and a role for Notch signaling. Physiol Genomics. 2009;36(2):69–78. 10.1152/physiolgenomics.90318.2008 18984672 PMC2636923

[25] Liu H, Kennard S, Lilly B. NOTCH3 expression is induced in mural cells through an autoregulatory loop that requires endothelial-expressed JAGGED1. Circ Res. 2009;104(4):466–75. 10.1161/CIRCRESAHA.108.184846 19150886 PMC2747310

[26] Kayamori K, Sakamoto K, Nakashima T, Takayanagi H, Morita K, Omura K, et al Roles of interleukin-6 and parathyroid hormone-related peptide in osteoclast formation associated with oral cancers: significance of interleukin-6 synthesized by stromal cells in response to cancer cells. Am J Pathol. 2010;176(2):968–80. 10.2353/ajpath.2010.090299 20035059 PMC2808100

[27] Momose F, Araida T, Negishi A, Ichijo H, Shioda S, Sasaki S. Variant sublines with different metastatic potentials selected in nude mice from human oral squamous cell carcinomas. J Oral Pathol Med. 1989;18(7):391–5. 2585303 10.1111/j.1600-0714.1989.tb01570.x

[28] Hetheridge C, Mavria G, Mellor H. Uses of the in vitro endothelial-fibroblast organotypic co-culture assay in angiogenesis research. Biochem Soc Trans. 2011;39(6):1597–600. 10.1042/BST20110738 22103493

[29] Terrinoni A, Serra V, Bruno E, Strasser A, Valente E, Flores ER, et al Role of p63 and the Notch pathway in cochlea development and sensorineural deafness. Proc Natl Acad Sci U S A. 2013;110(18):7300–5. 10.1073/pnas.1214498110 23589895 PMC3645580

[30] Pannequin J, Bonnans C, Delaunay N, Ryan J, Bourgaux JF, Joubert D, et al The wnt target jagged-1 mediates the activation of notch signaling by progastrin in human colorectal cancer cells. Cancer Res. 2009;69(15):6065–73. 10.1158/0008-5472.CAN-08-2409 19622776

[31] Lau TS, Chung TK, Cheung TH, Chan LK, Cheung LW, Yim SF, et al Cancer cell-derived lymphotoxin mediates reciprocal tumour-stromal interactions in human ovarian cancer by inducing CXCL11 in fibroblasts. J Pathol. 2014;232(1):43–56. 10.1002/path.4258 24014111

[32] Bergers G, Song S. The role of pericytes in blood-vessel formation and maintenance. Neuro Oncol. 2005;7(4):452–64. 16212810 10.1215/S1152851705000232 PMC1871727

[33] Gokcinar-Yagci B, Uckan-Cetinkaya D, Celebi-Saltik B. Pericytes: Properties, Functions and Applications in Tissue Engineering. Stem Cell Rev. 2015;11(4):549–59. 10.1007/s12015-015-9590-z 25865146

[34] van Dijk CG, Nieuweboer FE, Pei JY, Xu YJ, Burgisser P, van Mulligen E, et al The complex mural cell: pericyte function in health and disease. Int J Cardiol. 2015;190:75–89. 10.1016/j.ijcard.2015.03.258 25918055

[35] Gerhardt H, Betsholtz C. Endothelial-pericyte interactions in angiogenesis. Cell Tissue Res. 2003;314(1):15–23. 12883993 10.1007/s00441-003-0745-x

[36] Sainson RC, Harris AL. Regulation of angiogenesis by homotypic and heterotypic notch signalling in endothelial cells and pericytes: from basic research to potential therapies. Angiogenesis. 2008;11(1):41–51. 10.1007/s10456-008-9098-0 18256896

[37] Zhang P, Yan X, Chen Y, Yang Z, Han H. Notch signaling in blood vessels: from morphogenesis to homeostasis. Sci China Life Sci. 2014;57(8):774–80. 10.1007/s11427-014-4716-0 25104449

[38] Liu H, Zhang W, Kennard S, Caldwell RB, Lilly B. Notch3 is critical for proper angiogenesis and mural cell investment. Circ Res. 2010;107(7):860–70. 10.1161/CIRCRESAHA.110.218271 20689064 PMC2948576

[39] Hellstrom M, Phng LK, Hofmann JJ, Wallgard E, Coultas L, Lindblom P, et al Dll4 signalling through Notch1 regulates formation of tip cells during angiogenesis. Nature. 2007;445(7129):776–80. 17259973 10.1038/nature05571

[40] Kangsamaksin T, Tattersall IW, Kitajewski J. Notch functions in developmental and tumour angiogenesis by diverse mechanisms. Biochem Soc Trans. 2014;42(6):1563–8. 10.1042/BST20140233 25399571

[41] Neves J, Abello G, Petrovic J, Giraldez F. Patterning and cell fate in the inner ear: a case for Notch in the chicken embryo. Dev Growth Differ. 2013;55(1):96–112. 10.1111/dgd.12016 23252974

[42] Manderfield LJ, High FA, Engleka KA, Liu F, Li L, Rentschler S, et al Notch activation of Jagged1 contributes to the assembly of the arterial wall. Circulation. 2012;125(2):314–23. 10.1161/CIRCULATIONAHA.111.047159 22147907 PMC3260393

[43] Shi C, Qian J, Ma M, Zhang Y, Han B. Notch 3 protein, not its gene polymorphism, is associated with the chemotherapy response and prognosis of advanced NSCLC patients. Cell Physiol Biochem. 2014;34(3):743–52. 10.1159/000363039 25171754

[44] Ozawa T, Kazama S, Akiyoshi T, Murono K, Yoneyama S, Tanaka T, et al Nuclear Notch3 expression is associated with tumor recurrence in patients with stage II and III colorectal cancer. Ann Surg Oncol. 2014;21(8):2650–8. 10.1245/s10434-014-3659-9 24728738

[45] Mann CD, Bastianpillai C, Neal CP, Masood MM, Jones DJ, Teichert F, et al Notch3 and HEY-1 as prognostic biomarkers in pancreatic adenocarcinoma. PLoS One. 2012;7(12):e51119 10.1371/journal.pone.0051119 23226563 PMC3514220

[46] Park JT, Chen X, Trope CG, Davidson B, Shih Ie M, Wang TL. Notch3 overexpression is related to the recurrence of ovarian cancer and confers resistance to carboplatin. Am J Pathol. 2010;177(3):1087–94. 10.2353/ajpath.2010.100316 20671266 PMC2928943

[47] Zhang TH, Liu HC, Zhu LJ, Chu M, Liang YJ, Liang LZ, et al Activation of Notch signaling in human tongue carcinoma. J Oral Pathol Med. 2011;40(1):37–45. 10.1111/j.1600-0714.2010.00931.x 20819128

[48] Yen WC, Fischer MM, Axelrod F, Bond C, Cain J, Cancilla B, et al Targeting Notch signaling with a Notch2/Notch3 antagonist (tarextumab) inhibits tumor growth and decreases tumor-initiating cell frequency. Clin Cancer Res. 2015;21(9):2084–95. 10.1158/1078-0432.CCR-14-2808 25934888

