# Supplementary figures and images for: NOTCH3 Is Induced in Cancer-Associated Fibroblasts and Promotes Angiogenesis in Oral Squamous Cell Carcinoma
Source: PLoS One. 2016 Apr 28;11(4):e0154112. doi: 10.1371/journal.pone.0154112 (PMC4849776; doi:10.1371/journal.pone.0154112)

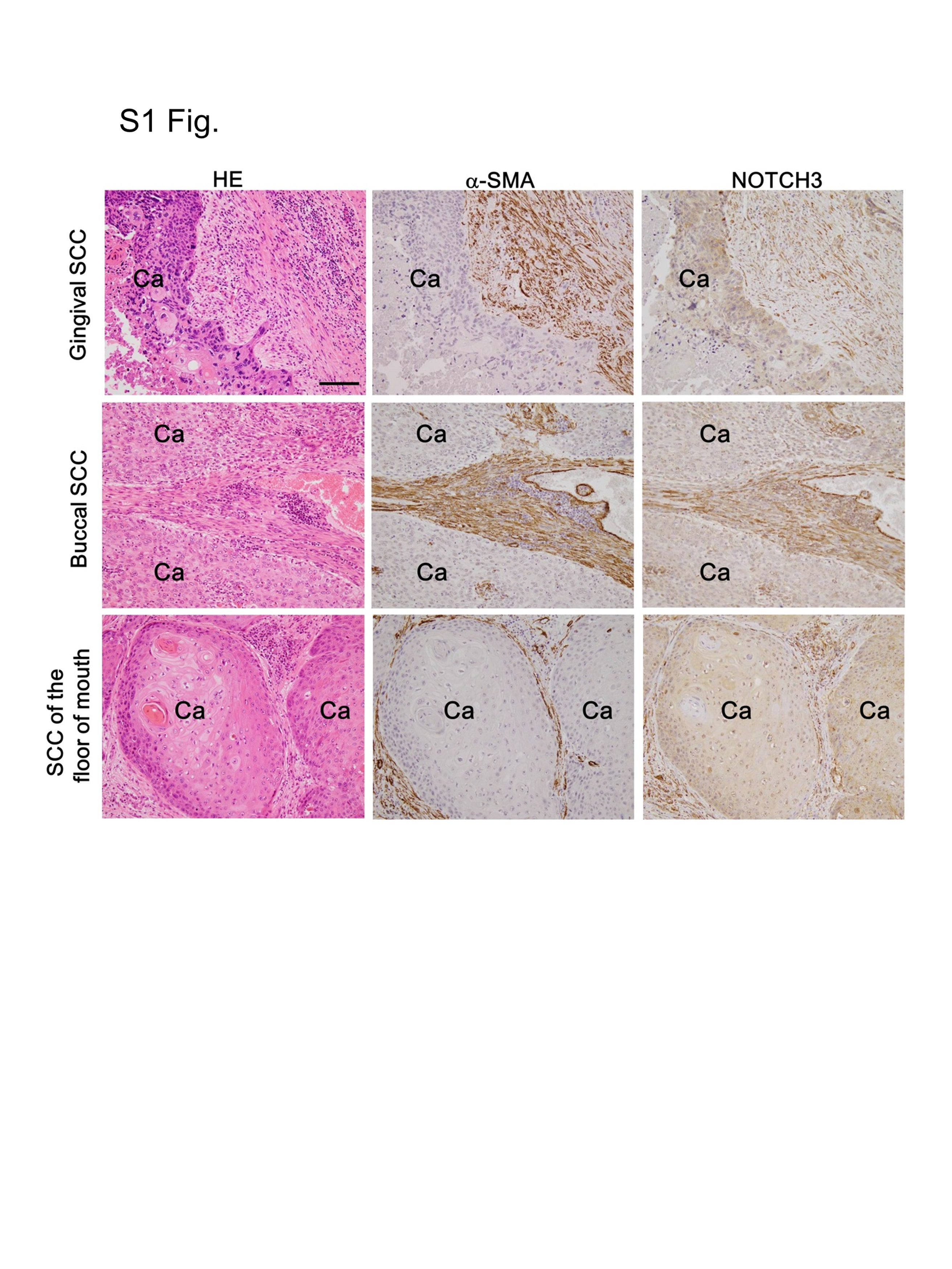

Supplement: S1 Fig — Immunohistochemical analyses for α-SMA and NOTCH3 in human gingival, buccal and floor of mouth SCC samples. Scale bar, 100μm. Ca, cancer cells. (TIF) [file pone.0154112.s001.tif]
